# Convalescent plasma treatment for SARS-CoV-2 infection: analysis of the first 436 donors in England, 22 April to 12 May 2020

**DOI:** 10.2807/1560-7917.ES.2020.25.28.2001260

**Published:** 2020-07-16

**Authors:** Heli Harvala, Jennifer Mehew, Matthew L Robb, Samreen Ijaz, Steven Dicks, Monika Patel, Nicholas Watkins, Peter Simmonds, Tim Brooks, Rachel Johnson, Robin Gopal, David J Roberts, Maria Zambon, the NHS Blood and Transplant Convalescent Plasma Testing Group, NHS Blood and Transplant Convalescent Plasma Testing Group, NHS Blood and Transplant Convalescent Plasma Testing Group, Stephen Thomas, Sheila MacLennan, Lise Estcourt, Su Brailsford, Hatice Baklan, Kate Tettmar, Jeremy Kellington, Joanne Sell, Gail Miflin

**Affiliations:** 1 Microbiology Services, NHS Blood and Transplant, London, United Kingdom; 2 Statistics and Clinical Studies, NHS Blood and Transplant, Bristol, United Kingdom; 3 Virology Reference Department, National Infection Service, Public Health England, Colindale Avenue, London, United Kingdom; 4 High Containment Microbiology, National Infection Service, Public Health England, Colindale Avenue, London, United Kingdom; 5 NHS Blood and Transplant, Cambridge, United Kingdom; 6 Nuffield Department of Medicine, University of Oxford, Oxford, United Kingdom; 7 Rare & Imported Pathogens Laboratory, Public Health England, Porton Down, United Kingdom; 8 NHS Blood and Transplant, Oxford, John Radcliffe Hospital, Oxford, United Kingdom; 9 Radcliffe Department of Medicine and BRC Haematology Theme, University of Oxford, John Radcliffe Hospital, Oxford, United Kingdom; 10 The members of the NHS Blood and Transplant Convalescent Plasma Testing Group are listed below

**Keywords:** SARS-CoV-2, convalescent plasma, neutralising antibodies, antibody response, symptomatic infection, high titre

## Abstract

Serological reactivity was analysed in plasma from 436 individuals with a history of disease compatible with COVID-19, including 256 who had been laboratory-confirmed with SARS-CoV-2 infection. Over 99% of laboratory-confirmed cases developed a measurable antibody response (254/256) and 88% harboured neutralising antibodies (226/256). Antibody levels declined over 3 months following diagnosis, emphasising the importance of the timing of convalescent plasma collections. Binding antibody measurements can inform selection of convalescent plasma donors with high neutralising antibody levels.

The emergence in China, at the end of 2019, of severe acute respiratory syndrome coronavirus 2 (SARS-CoV-2), a virus causing coronavirus disease (COVID-19), was followed by a rapid spread of the virus, leading to the announcement of a COVID-19 pandemic on 11 March 2020 [1,2]. Without an effective treatment or a vaccine, convalescent plasma therapy has been recommended to tackle COVID-19 associated morbidity and mortality [3,4]. Neutralising antibodies to SARS-CoV-2 in plasma collected from recovered patients is likely to support such therapy [5-7]. The timing and nature of immune response associated with SARS-CoV-2 infection is variable in recovering individuals, although seroconversion is typically detectable 14 days post infection [8-11]. Furthermore, higher neutralising antibody levels have been measured in older individuals [11,12] and those with more severe SARS-CoV-2 infections [10]. Here we analysed the performance of serological assays designed to detect antibodies against SARS-CoV-2 and assessed host factors associated with elevated neutralising antibody levels in order to improve donor selection.

## Collecting plasma samples

In England, the National Health Service (NHS) Blood and Transplant is collecting convalescent plasma from individuals with confirmed or suspected SARS-CoV-2 infection at least 28 days after the resolution of their symptoms, and donations containing a minimum neutralising antibody titre of 1:100 are provided for clinical use [13,14]. 

During the first weeks of convalescent plasma apheresis collections (22 April to 12 May), a total of 436 donations were obtained. Donors were aged between 17 and 65 years. Convalescent plasma was primarily collected from individuals for whom SARS-CoV-2 infection had been laboratory-confirmed by RT-PCR, but donations were also taken from individuals with self-reported previous suspected infection. Based on the NHS Blood and Transplant donation and NHS Digital diagnostic record matching, 256 convalescent plasma donors were identified as having had a previous laboratory-confirmed SARS-CoV-2 infection (256/436, 59%). The diagnosis had been made between 31 and 60 days before the donation, and fewer than 10% were known to have been hospitalised (22/256). Some of the remaining donors may also have had a laboratory-confirmed SARS-CoV-2 infection, but this could not be confirmed.

## Ethical statement

Signed donor consent was obtained for the purposes of clinical audit, to assess and improve the service and for research, and specifically to improve our knowledge of the donor population.

## Detection of antibodies and sample processing

All donations were tested for SARS-CoV-2 RNA by RT-PCR and antibodies. The presence of IgG antibodies in all plasma samples was assessed using a SARS-CoV-2 infected cell lysate ELISA assay and by Euroimmun ELISA (S1; PerkinElmer, London, United Kingdom), which uses the spike protein as antigen. Neutralising antibodies were detected using a microneutralisation assay as previously described [13]. Donations with a signal to cut-off (S/CO) ratio of 9.1 or higher in the Euroimmun assay were released for clinical use before microneutralisation assay results were available as this cut-off was previously shown to identify donations with a minimum neutralising antibody titre of 1:100 with a specificity of 100% [13].

## Evidence of past infection in plasma donors and antibody detection assays’ performance

Most convalescent plasma donors showed serological evidence of past SARS-CoV-2 infection, with 379 samples reactive in the virus lysate assay (86.9%), and 346 showing detectable IgG antibodies in the Euroimmun assay (79.4%) (Table). A total of 331 samples had detectable neutralising antibodies (75.9%).

**Table:** Results of Euroimmun IgG ELISA, live virus lysate total IgG antibody ELISA, and microneutralisation test for neutralising antibody detection, on plasma samples of donors recovered from self-reported or laboratory-confirmed SARS-CoV-2 infections (n = 436 plasma samples)

Samples	Total number	Euroimmun IgG ELISA			Live virus lysate ELISA			Neutralising antibody test		
		Reactive	Non-reactive	% Reactive	Reactive	Non-reactive	% Reactive	Detected	Not detected	% Detected
**All samples**	436	346	90	79.4	379	57	86.9	331	105	75.9
**Samples from confirmed cases**										
**All with confirmed diagnosis**	256	232	24	90.6	254	2	99.2	226	32	88.2
**30–40** **days post diagnosis**	91	83	8	91.2	91	0	100	79	12	86.8
**40–50** **days post diagnosis**	123	115	8	93.5	123	0	100	112	11	91.1
**>** **50** **days post diagnosis**	42	34	8	81.0	40	2	95.2	35	7	83.3

SARS-CoV-2: severe acute respiratory syndrome coronavirus 2.

The sensitivity of immunoassays was determined based on donors with a previous laboratory-confirmed SARS-CoV-2 infection, all sampled at least 30 days after diagnosis (n = 256). SARS-CoV-2 antibodies were detected in 254/256 donors by virus lysate assay (sensitivity of 99.2%) and in 232/256 by Euroimmun (90.6%) (Table). Each assay showed a decrease in detection rates over time elapsed from diagnosis. Neutralising antibodies were detected in 226/256 donors (88.2%), from which eight were notably negative by Euroimmun assay.

These findings confirm that most individuals with symptomatic SARS-CoV-2 infection develop measurable antibody responses, although the sensitivity of the assays evaluated is variable. The native virus ELISA assay format has been previously used for Middle East respiratory syndrome coronavirus (MERS-CoV) detection and shown to have a similar sensitivity, likely due to the presence of multiple viral antigens derived from infected cell cultures [15]. The low sensitivity of Euroimmun assay used here is in keeping with a recently reported sensitivity rate of 70.7% [16], albeit based on low sample numbers and with only 10% tested (6/62) after 30 days of disease onset.

## Host factors and neutralising antibody levels in plasma

**Figure 1:**
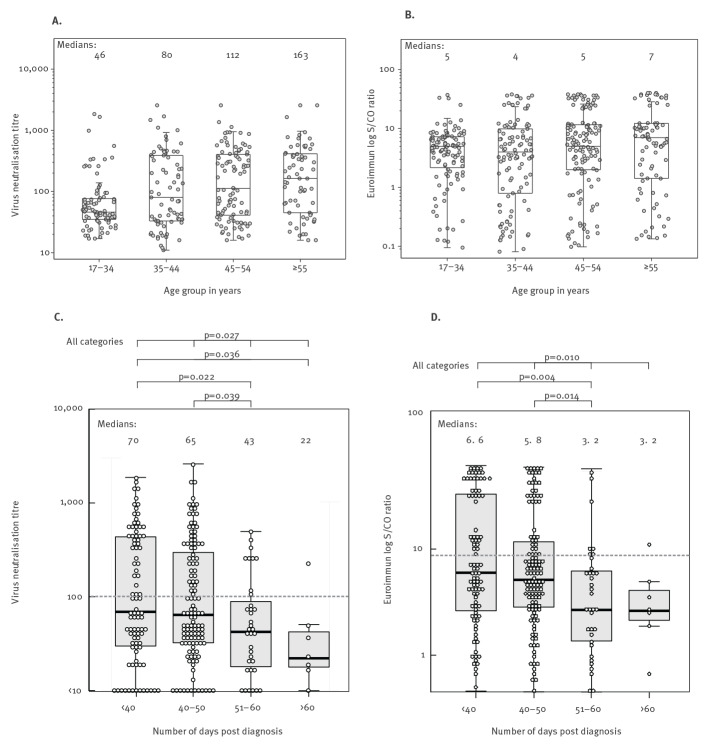
Neutralising antibody titres and serological reactivity in Euroimmun IgG ELISA of plasma samples from donors recovered from COVID-19, relative to (A,B) the age of donors and (C,D) the time of diagnosis (n = 436 plasma samples)

Neutralising antibody levels varied, with geometric mean titre (GMT) 1:333 (range < 1:10–1:2,560). Titres of 1:100 or higher, aimed for clinical use, were measured in 34% of donations (147/436). The highest levels of neutralising antibodies were found in donors hospitalised with laboratory-confirmed SARS-CoV-2 infection (data not shown, n = 22), those who were older (Figure 1A) and those who donated < 60 days from diagnosis (Figure 1C). Consistent with the lower detection rates, there was evidence for a significant decline in neutralising antibody levels over time. Median neutralising antibody titre significantly decreased from 1:70 in those donating within 40 days from diagnosis to 1:43 and 1:22 in those donating at least 50 days (Kruskal–Wallis test; p = 0.022) or 60 days (Kruskal–Wallis test; p = 0.027) from diagnosis (Figure 1C). Similar findings were previously demonstrated in other studies on convalescent plasma donors where the proportion of high titre donors (antibody level 1:512 or above) decreased from 52% on days 31–40 post symptom onset to 28% on days 41–53 [12], and decreasing neutralising antibody levels have also been noted in SARS-CoV-2 infected hospitalised patients [17].

COVID-19: coronavirus disease; S/CO: signal to cut-off ratio.

A significant decline in virus neutralisation titre (p = 0.027) and Euroimmun reactivity (p = 0.010) was observed over time by Kruskal–Wallis test.

Similar to the neutralising antibody titres, a decrease was observed in Euroimmun S/CO by age (Figure 1B) and as time post diagnosis increased (Figure 1D), the latter being significant by Kruskal–Wallis test (p = 0.010).

## Predicting samples potentially suitable for convalescent plasma therapy using binding antibody titres

**Figure 2:**
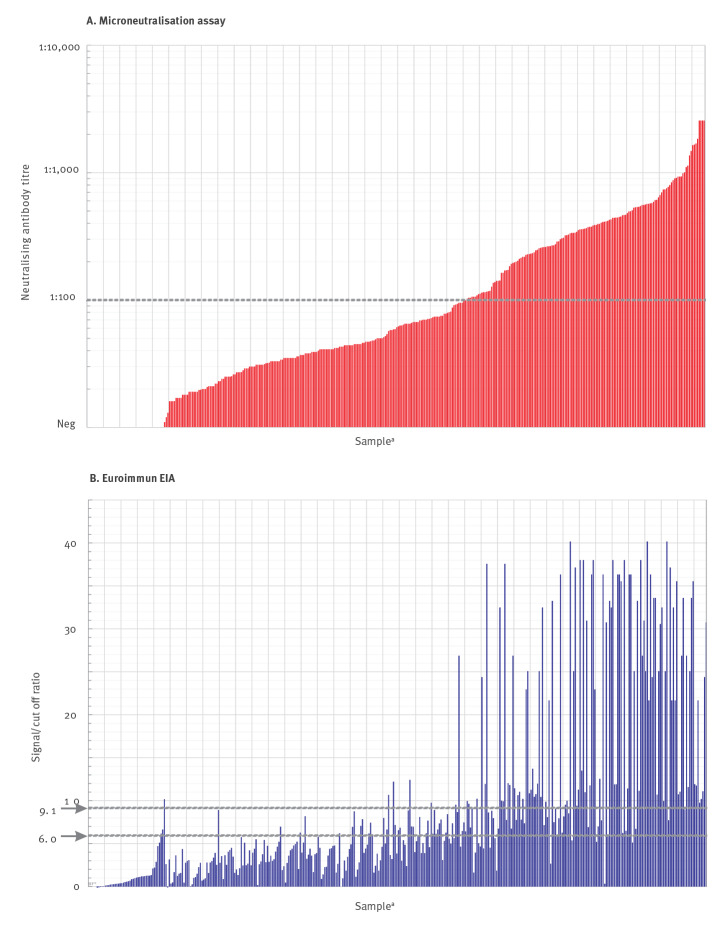
(A) Virus neutralising antibody titres and (B) reactivity in Euroimmun assay, for plasma samples from donors recovered from confirmed or suspected COVID-19 (n = 436 plasma samples)

COVID-19: coronavirus disease; EIA: enzyme immunoassay; neg: negative.

^a^ In all graphs, samples were ordered by virus neutralising antibody titres.

**Figure 3:**
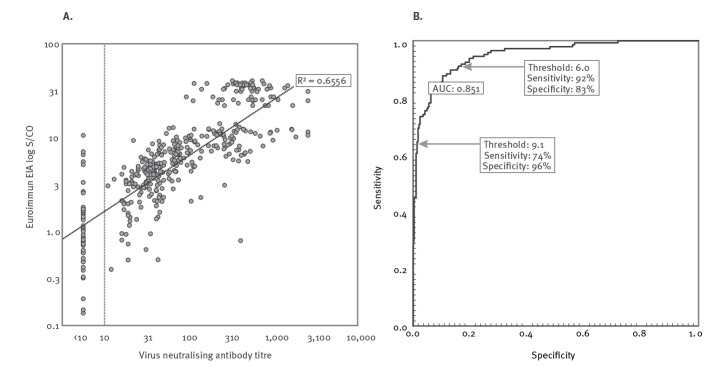
(A) Correlations between virus neutralising antibody titres and reactivities in Euroimmun ELISAs for plasma samples from donors recovered from confirmed or suspected COVID-19 and (B) receiver operating characteristic (ROC) analysis (n = 436 plasma samples)

COVID-19: coronavirus disease; EIA: enzyme immunoassay; ROC: receiver operating characteristic; S/CO: signal to cut-off ratio; AUC: the area under curve.

Antibody reactivities in Euroimmun assay showed associations with neutralising antibody titres (Figure 2,3) and were strongly predictive of neutralising antibody titres by linear regression using log transformed values (R^2^=0.6556, p<0.0001; Figure 3A). By receiver operating characteristic (ROC) analysis, we investigated the sensitivity and specificity of the Euroimmun IgG assay for prediction of neutralising antibody titres of 1:100 or greater (Figure 3B). Lowering the currently used cut-off value of 9.1 to 6.0 would increase the number of high titre donations identified from 74% to 92% but would also reduce specificity, with false identification of units below the 1:100 threshold increasing from 4% to 17%. This would translate to the marginal reduction of the median neutralisation titre from 1:375 (90^th^ percentile: 1:65 to 1:1,658) to 1:261 (90^th^ percentile: 1:39 to 1:1,133). Based on these calculations, and in the absence of scalable neutralisation antibody test, we are now accepting all convalescent plasma donations with a minimum cut-off ratio of 6.0 in the Euroimmun assay for clinical use.

Comparison of neutralising antibody titres of the 436 convalescent plasma donor samples with reactivity in Euroimmun assay. Dotted lines show thresholds for therapeutic use in the neutralisation antibody assay (1:100) and Euroimmun assays (9.1 and 6.0).

A. Scatter plots of neutralising antibody titres of test samples with reactivities in Euroimmun assay. A line of best fit was estimated by linear regression using log-transformed values for the virus neutralising antibody and Euroimmun assays (p = 2 x 10^-67^).

B. ROC analysis of serology assays predicting virus neutralising antibody titres of ≥ 1:100 (n = 436).

## Conclusions

The study findings support the prioritisation of donation collection from only those with a laboratory-confirmed SARS-CoV-2 infection. In the study sample of 436 donations, 57 samples (13%) were negative in all three serological assays. Evidence of a laboratory-confirmed SARS-CoV-2 infection was found only for two of these seronegative donors, compared with 254 in the 379 seropositive donations (67%). With regard to the two initial laboratory-confirmed infections, subsequently testing negative by serological assay, we recognise that individuals with very mild infections may fail to develop a measurable immunoresponse. For the 55 seronegative donors with self-reported, non-prior-laboratory-confirmed COVID-19, it is also possible that they simply did not have SARS-CoV-2 infection, given the lack of specificity of diagnosis based on symptoms only. Given the currently limited sensitivity and specificity of antibody tests for SARS-CoV-2 by conventional diagnostic standards [16-20], reporting of results to such donors needs to be undertaken very carefully and the test limitations explained.

In conclusion, most individuals with previously laboratory-diagnosed SARS-CoV-2 infection develop measurable antibody responses and also develop neutralising antibodies. A self-diagnosed infection is not a desirable selection criterion for convalescent plasma donors. Neutralising antibody levels declined within the first 3 months following diagnosis, which suggests the collection of convalescent plasma with high neutralising antibody may be optimum within a short time window. Finally, the study indicates that commercial ELISA can perform effectively as surrogate assays for predicting neutralising antibody titres and represent a streamlined and rapid way to guide convalescent plasma donor selection.
