# Correction for Euro Surveill. 2020;25(28)

**DOI:** 10.2807/1560-7917.ES.2021.26.12.210325d

**Published:** 2021-03-25

**Authors:** Eurosurveillance editorial team

**Affiliations:** 1 European Centre for Disease Prevention and Control (ECDC), Stockholm, Sweden

In the article ‘Convalescent plasma treatment for SARS-CoV-2 infection: analysis of the first 436 donors in England, 22 April to 12 May 2020’ by Harvala et al., published on 16 July 2020, a statement regarding funding was added to the acknowledgements’ section on 25 March 2021, at the request of the authors.

